# Trends in dietary patterns over the last decade and their association with long-term mortality in general US populations with undiagnosed and diagnosed diabetes

**DOI:** 10.1038/s41387-023-00232-8

**Published:** 2023-04-19

**Authors:** Sheng Yuan, Jining He, Shaoyu Wu, Rui Zhang, Zheng Qiao, Xiaohui Bian, Hongjian Wang, Kefei Dou

**Affiliations:** 1 Cardiometabolic Medicine Center, Department of Cardiology, Fuwai Hospital, National Center for Cardiovascular Diseases, Chinese Academy of Medical Sciences and Peking Union Medical College, Beijing, China; 2 State Key Laboratory of Cardiovascular Disease, Beijing, China; 3 National Clinical Research Center for Cardiovascular Diseases, Beijing, China

**Keywords:** Epidemiology, Type 2 diabetes, Epidemiology

## Abstract

**Background:**

Dietary management plays an important role in diabetes care, while the trends in dietary patterns over the last decade in US adults with diagnosed and undiagnosed diabetes remain unknown. This study aims to estimate the dietary patterns over the last decade by baseline diabetes diagnoses and explore their association with long-term prognosis.

**Methods:**

Participants’ data were extracted from the National Health and Nutrition Examination Survey (NHANES) 2007–2018, which were divided into three groups according to the diabetes diagnosis: without diabetes, undiagnosed diabetes, and diagnosed diabetes. Healthy eating index (HEI) and dietary inflammatory index (DII) were used to evaluate dietary patterns. Survival analyses were adopted to estimate the association between HEI/DII scores and long-term all-cause mortality and cause-specific mortality.

**Results:**

The prevalence of diabetes was increasing among US adults over the last decade. HEI scores of all three groups presented a downward trend in recent years. Participants with undiagnosed diabetes (weighted mean: 50.58, 95% CI: 49.79, 51.36) got significantly lower HEI score in comparison to participants with diagnosed diabetes (weighted mean: 51.59, 95% CI: 50.93, 52.25). Compared with participants without diabetes, participants in the undiagnosed or diagnosed diabetes group had higher DII scores, indicating a higher dietary inflammatory potential. Survival analysis found a significant association between HEI scores and all-cause mortality and death of heart diseases. Similar correlation was observed in DII scores.

**Conclusions:**

Along with the growth in diabetes prevalence in the US, dietary management of people with diabetes is decreasing. The management of US adults’ diets needs special attention, and dietary inflammatory potential may be considered in the dietary intervention.

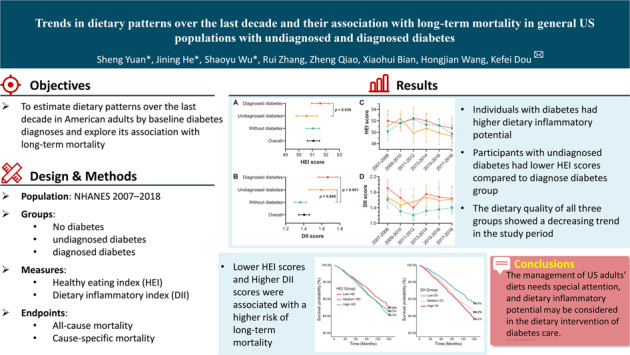

## Introduction

Diabetes mellitus and its complications remain a leading cause of death and pose a great economic burden worldwide [1, 2]. According to the International Diabetes Federation’s estimation, 536.6 million adults (20–79 years old) suffered from diabetes, with that number expected to rise to 783.2 million by 2045 [3]. Another source of concern is the high proportion of undiagnosed diabetes (44.7%, 239.7 million) [4]. The unawareness of diabetes results in a lack of appropriate and timely care. It’s urgent to know the characteristics of patients with undiagnosed diabetes and enroll them in standard diabetes care to prevent or delay the development of complications.

Nutrient intake and dietary patterns have been proven to be related with the risk of diabetes, which is also a significant part of lifestyle intervention in diabetes management [5]. For instance, the Mediterranean diet has been reported to be strongly correlated with a lower risk of diabetes [6]. On the contrary, some foods or characteristics such as processed red meat, sugar-sweetened beverages, and high dietary inflammatory potential were associated with an increased risk of diabetes [7–9].

The healthy eating index (HEI) is a classic assessment of overall dietary quality, which was developed by the US Department of Agriculture to measure the adherence to Dietary Guidelines for Americans [10]. Previous research demonstrated that higher HEI scores were associated with a lower risk of cardiovascular disease (CVD) and diabetes, but its association with the prognosis of diabetes remains to be investigated [11–15]. Besides, dietary inflammatory index (DII) was another dietary index that was developed to measure the dietary inflammatory potential [16]. High dietary inflammatory potential, which is reflected by high DII scores, was proven to be associated with an increased risk of diabetes [17, 18].

Despite the fact that the link between diet and diabetes incidence is well established, there is currently little data on the relationship between dietary patterns and the prognosis of patients with diabetes. Moreover, there is no report on the dietary habits of people with undiagnosed diabetes. Our study used the HEI and DII scores to quantify trends of dietary patterns in general US adults over the last decade by diabetes diagnoses, as well as its association with individual prognosis, which may contribute to the lifestyle intervention of patients with diabetes, particularly those with undiagnosed diabetes.

## Methods

### Setting and participants

The National Health and Nutrition Examination Survey (NHANES), conducted by the American National Center for Health Statistics, is a periodical program that adopts a complex, multistage, probability sampling design to recruit individuals that are representative of the general US population [19]. Participants of this work come from the NHANES 2007–2018 cycle. We included individuals who met the following criteria: (1) age ≥20 years old; (2) participants without pregnancy; (3) Participants with available 24-hour dietary interview data. The survival analysis extracted participants from the NHANES 2007–2014 cycle and excluded individuals without follow-up information in combination with other prespecified inclusion criteria.

This retrospective cohort study was carried out in accordance with the Strengthening the Reporting of Observational Studies in Epidemiology reporting guideline for cohort studies [20].

### HEI and DII calculation

Original dietary data were collected from NHANES dietary interview, which contains a dietary recall about the food, beverages, and supplements that consumed during the 24-hour prior to the interview and has been validated elsewhere [21].

HEI-2015 is the latest version of HEI, which consists of 13 food parameters including total vegetables, greens & beans, total fruits, whole fruits, whole grains, dairy, total protein foods, seafood & plant proteins, fatty acids, sodium, refined grains, saturated fats, added sugars. The total and component HEI scores were calculated following the four steps of the HEI scoring algorithm on the website and a simple HEI scoring algorithm was adopted to calculate the total and component HEI scores [22]. According to the DII calculating method reported by N. Shivappa et al. [16], we extracted 28 food parameters from the 24-hour dietary recall, including protein, carbohydrates, total fat, cholesterol, saturated fat, polyunsaturated fatty acids (PUFAs), monounsaturated fatty acids (MUFAs), alcohol, niacin, vitamin A, riboflavin, thiamin, vitamin B6, vitamin B12, vitamin C, vitamin D, vitamin E, fiber, Mg, Fe, Zinc, Selenium, β-carotene, folic acid, n-3 fatty acids, n-6 fatty acids, caffeine, and energy. The process of DII calculation was presented in previous work [23]. Overall, a higher and positive DII score represents a pro-inflammatory dietary potential, while a lower and negative DII score means an anti-inflammatory dietary potential. The intact DII score system consists of 45 food parameters, but it has been demonstrated that DII calculated from less than 30 food parameters remained its predictive value [24, 25].

### Outcome and covariates

The follow-up was ended on December 31, 2019. The primary endpoint was all-cause death, and the secondary endpoints included death of malignant neoplasms, death of heart disease, death of chronic lower respiratory diseases, and death of cerebrovascular diseases, all of which were selected according to the leading causes of death in the United States and were extracted from the National Death Index (NDI) records [26, 27]. The definition of cause-specific death was based on the International Classification of Diseases -10 codes (Malignant neoplasms: C00-C97; heart disease: I00-I09, I11, I13, I20-I51; chronic lower respiratory diseases: J40-J47; cerebrovascular diseases: I60-I69).

Participants were diagnosed as diabetes if they met one of the following diagnostic criteria: (1) a self-reported physician diagnosis of diabetes; (2) fasting glucose ≥7.0 mmol/L; (3) 2-hour plasma glucose levels in oral glucose tolerance test ≥11.1 mmol/L; (4) glycated hemoglobin A_1c_ (HbA_1c_) ≥6.5%; (5) use of diabetes medication or insulin. Participants who met one of 2–5 criteria but without a self-reported physician diabetes diagnosis were divided into the undiagnosed diabetes group, while participants who met the first criterion were grouped into diagnosed diabetes group. The definition of hyperlipidemia was: (1) total cholesterol ≥200 mg/dL; (2) low-density lipoprotein-cholesterol (LDL-C) ≥ 130 mg/dL; (3) high density lipoprotein-cholesterol (HDL-C) < 40 mg/dL in men and 50 mg/dL in women; (4) triglyceride ≥ 150 mg/dL; (5) use of cholesterol-lowering drugs. The diagnostic criteria for hypertension were (1) a self-reported diagnosis of hypertension from a physician or other health professional; (2) systolic blood pressure ≥140 mmHg and/or diastolic blood pressure ≥90 mmHg (at least 3 times); (3) use of antihypertensive drugs. The moderate or heavy drink was defined as: (1) ≥3 drinks per day for men or ≥2 drinks per day for women; (2) binge drinking ≥2 days per month. Binge drinking was defined as ≥5 drinks at a time for men or ≥4 drinks for women [28].

### Statistical analysis

Due to the multistage complex design of NHANES, all analyses in this work have incorporated oversampling, clustering, and stratification to estimate the representative statistics of the general US population [19]. According to the type of variables, baseline characteristics were listed as weighted mean and 95% confidence interval (CI) (continuous variables) or weighted proportions (categorical variables). Comparison of parameters among three groups (without diabetes, with undiagnosed diabetes, and with diagnosed diabetes) was performed by weighted generalized linear regression models (continuous variable), weighted chi-square test (categorical variable), or weighted logistic regression models (categorical variable). The radar plot was plotted to show the distribution of HEI component scores, which was generated by divide the component score by the maximum score of that component. The max scores of each HEI food parameter were listed in Supplementary Table S1.

Participants were equally divided into 3 groups according to their HEI or DII scores: low HEI (0 ≤ HEI ≤ 44.4), medium HEI (44.4 < HEI ≤ 56.8), high HEI (56.8 < HEI ≤ 96.0), and low DII (−5.28 ≤ DII ≤ 0.81), medium DII (0.81 < DII ≤ 2.65), high DII (2.65 < DII ≤ 5.47). To investigate whether dietary patterns correlate with the long-term mortality of the general US population, we performed age-adjusted weighted Cox proportional hazard regression models and included HEI or DII as continuous or categorical variables respectively. Furthermore, the weighted Cox regression models have been adjusted for age, sex, educational level, body mass index (BMI), smoking, hypertension, hyperlipidemia, diabetes diagnosis, and alcohol consumption. *P* for interaction was calculated by baseline diabetes diagnosis.

We considered a two-sided *p* value < 0.05 as statistically significant. All statistics were conducted using the R software version 4.1.2 (R Foundation for Statistical Computing, Vienna, Austria).

## Results

Following the prespecified inclusion criteria, a total of 30 442 individuals were included in our study, which represented 224 220 469 general US population after weighted transformation (supplementary Fig. S1).

### Diabetes prevalence and general information by baseline diabetes diagnoses

The weighted proportion of individuals with diabetes was 14.47%, of which 4.54% was undiagnosed diabetes. As shown in supplementary Figure S2, diabetes prevalence in the United States has steadily increased over the last decade, reaching 15.55% in the 2017–2018 cycle. Table 1 listed the basic characteristics of participants with different diabetes diagnoses. Compared with participants without diabetes, the characteristics of patients with diabetes were older, higher BMI and waist, lower educational level, lower level of HDL-C, higher level of SBP, HbA1c, triglycerides, and a higher proportion of chronic disease such as hypertension and hyperlipidemia. We have also noticed a lower percentage of never smoker and current smoker, as well as a lower percentage of moderate or heavy drinkers in participants with diabetes. Furthermore, the total cholesterol and LDL-C level of participants with diagnosed diabetes were significantly lower compared to no diabetes group and undiagnosed diabetes group.

**Table 1:** Baseline characteristics according to diabetes diagnoses.

	Total (*n* = 30 442)	Without diabetes (*n* = 24 537)	Undiagnosed diabetes (*n* = 1804)	Diagnosed diabetes (*n* = 4101)	*p*
Age (years)	47.73 (47.24, 48.22)	45.71 (45.20, 46.22)	57.94 (57.03, 58.85)	60.44 (59.83, 61.06)	<0.001
Male (%)	48.68	48.33	49.50	51.38	0.081
BMI (kg/m^2^)	29.09 (28.92, 29.26)	28.44 (28.27, 28.61)	32.85 (32.32, 33.37)	32.99 (32.60, 33.38)	<0.001
Waist (cm)	99.32 (98.88, 99.75)	97.47 (97.03, 97.92)	109.37 (108.19, 110.56)	110.58 (109.80, 111.35)	<0.001
Ethnicity (%)					<0.001
Mexican American	8.61	8.38	10.65	9.64	
Non-Hispanic black	11.29	10.75	12.94	15.26	
Non-Hispanic white	66.37	67.32	61.32	60.50	
Other Hispanic	5.81	5.76	7.10	5.70	
Other race	7.92	7.80	7.99	8.90	
Educational level (%)					<0.001
Less than high school	15.63	14.32	21.59	24.14	
High school or equivalent	23.35	22.93	26.65	25.43	
College or above	61.03	62.75	51.75	50.43	
Smoking (%)					<0.001
Never smoker	55.31	56.27	51.96	48.59	
Former smoker	24.76	23.12	32.73	35.25	
Current smoker	19.93	20.61	15.32	16.16	
Moderate or heavy drink (%)	36.43	38.65	29.61	20.45	<0.001
SBP (mmHg)	122.81(122.43, 123.20)	121.55 (121.19, 121.91)	130.53 (129.06, 131.99)	130.42 (129.50, 131.34)	<0.001
Hypertension (%)	37.15	31.69	58.13	74.53	<0.001
Hyperlipidemia (%)	68.68	65.45	87.39	87.91	<0.001
HbA1c (%)	5.64 (5.62, 5.66)	5.40 (5.39, 5.41)	6.61 (6.51, 6.71)	7.30 (7.24, 7.37)	<0.001
Total cholesterol (mg/dL)	193.80 (192.87, 194.74)	195.08 (194.08, 196.09)	198.90 (196.00, 201.79)	180.49 (178.48, 182.50)	<0.001
HDL-C (mg/dL)	53.26 (52.85, 53.66)	54.23 (53.82, 54.65)	48.30 (47.27, 49.33)	47.12 (46.43, 47.81)	<0.001
LDL-C (mg/dL)	114.29 (113.29, 115.28)	115.99 (114.96, 117.02)	116.77 (113.48, 120.06)	98.44 (96.45, 100.43)	<0.001
Triglycerides (mg/dL)	125.11 (122.52, 127.71)	117.54 (115.13, 119.95)	158.68 (150.91, 166.45)	164.05 (153.60, 174.49)	<0.001

Continuous variables were presented as weighted mean and 95% confidence interval. *BMI* body mass index, *DBP* diastolic blood pressure, *HDL-C* high-density lipoprotein cholesterol, *LDL-C* low-density lipoprotein cholesterol, *SBP* systolic blood pressure.

### Dietary patterns of participants with different diabetes diagnoses

**Fig. 1:**
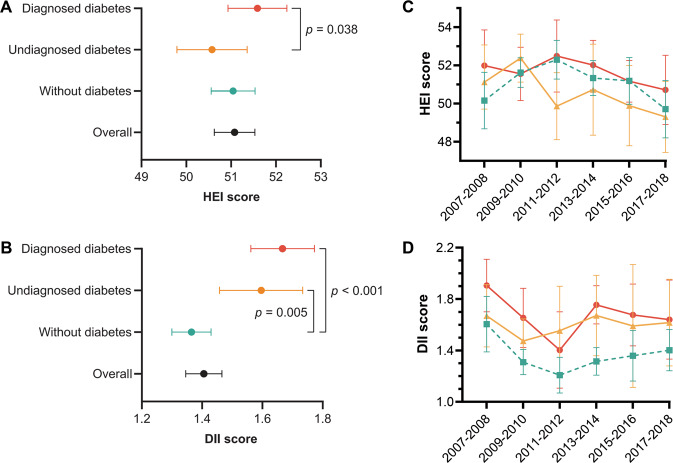
Comparison of dietary scores of general Americans by baseline diabetes diagnoses. **A** Comparison of the overall HEI score according to different diabetes diagnoses; **B** Comparison of the overall DII score according to different diabetes diagnoses; **C** US Trends in HEI over the last decade by baseline diabetes diagnoses; **D** US Trends in DII over the last decade by baseline diabetes diagnoses. The dots presented the weighted mean and the lines were the 95% confidence interval. The diagnosed diabetes group was depicted in red, while the groups with undiagnosed diabetes group and no diabetes were plotted in yellow and green, respectively. DII dietary inflammatory index, HEI healthy eating index.

The weighted mean of HEI score of the overall population was 51.08 (95% CI: 50.63, 51.53). As shown in Fig. 1, participants with undiagnosed diabetes (weighted mean: 50.58, 95% CI: 49.79, 51.36) got significantly lower HEI score in comparison to participants with diagnosed diabetes (weighted mean: 51.59, 95% CI: 50.93, 52.25). On the other hand, compared with participants without diabetes (weighted mean: 1.37, 95% CI: 1.30, 1.43), participants in the undiagnosed (weighted mean: 1.60, 95% CI: 1.46, 1.73) or diagnosed (weighted mean: 1.67, 95% CI: 1.56, 1.77) diabetes group got higher DII scores, which means a higher dietary inflammatory potential. Moreover, the HEI scores of all three groups presented a downward trend in recent years (Fig. 1C). The HEI scores of individuals with diagnosed diabetes were higher or similar compared with no diabetes group, which may indicate an effective dietary management in diabetes care. However, the HEI scores of participants with undiagnosed diabetes were lower than participants with diagnosed diabetes or without diabetes since 2011. In recent years, the DII scores of participants with diagnosed or undiagnosed diabetes were comparable, which were both significantly higher than participants without diabetes (Fig. 1D).

### Component analysis of healthy eating index by baseline diabetes diagnoses

**Fig. 2:**
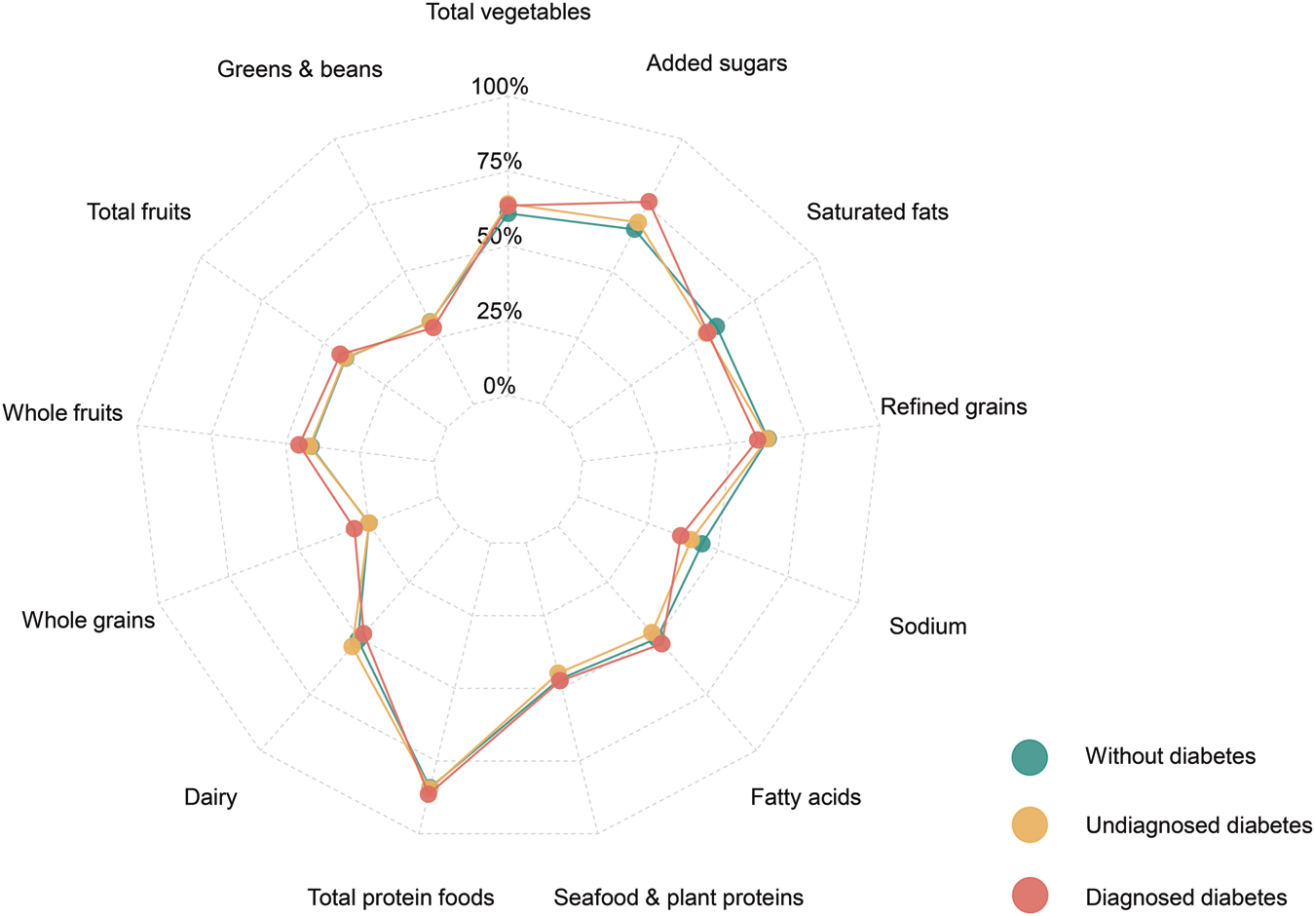
Characteristics of healthy eating index components by baseline diabetes diagnoses. Each component score was divided by the maximum score of that component.

As shown in Fig. 2, compared with no diabetes group, participants with diagnosed diabetes got higher scores in several food parameters like total vegetables, total fruits, whole fruits, whole grains, total protein foods, fatty acids, and added sugars, but significantly lower scores in parameters including dairy, sodium, refined grains, and saturated fats (Supplementary Table S2). Notably, participants with undiagnosed diabetes got lower scores in whole grains, fatty acids, and added sugars and higher scores in dairy, sodium, and refined grains. In the past decade, the scores of fatty acids and saturated fats in participants with undiagnosed diabetes showed a gradual decline (Supplementary Figure S3). Individuals with diagnosed diabetes consistently performed poorly on food parameters including sodium, dairy, and refined grains.

### Association between dietary patterns and long-term mortality

**Fig. 3:**
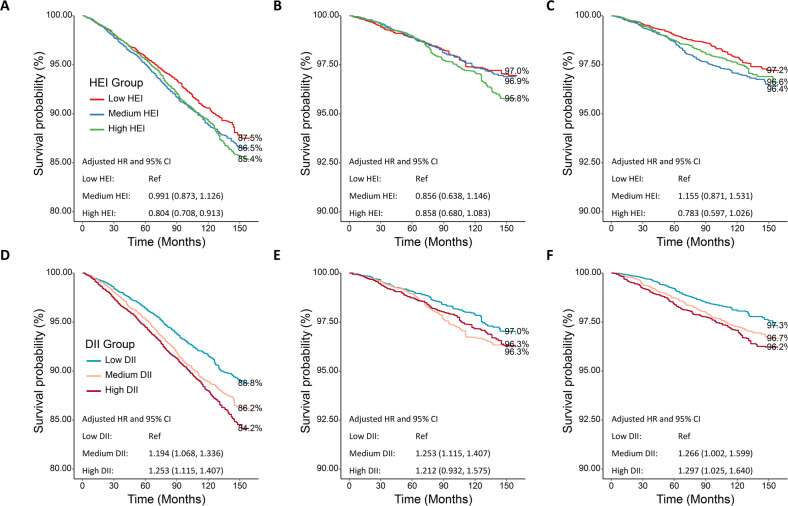
Association between dietary patterns and long-term mortality. **A** Association between HEI and all-cause mortality; **B** Association between HEI and death of malignant neoplasms; **C** Association between HEI and death of heart diseases; **D** Association between DII and all-cause mortality; **E** Association between DII and death of malignant neoplasms; **F** Association between DII and death of heart diseases. CI confidence interval, DII dietary inflammatory index, HEI healthy eating index; HR: hazard ratio.

To confirm the association between dietary patterns and the prognosis of the general US adults, weighted Cox regression models were conducted to estimate the association between two dietary indexes and long-term mortality. The included participants came from NHANES 2007–2014, which were listed in Supplementary Figure S4. The median follow-up time was 8.6 years. Overall, the weighted incidence of all-cause mortality was 9.36%, 2.31% for death of malignant neoplasms, 2.21% for death of heart diseases, 0.58% for death of chronic lower respiratory diseases, and 0.46% for death of cerebrovascular diseases. As shown in Fig. 3, supplementary Figure S5, and supplementary Table S3, higher DII scores were associated with higher all-cause mortality (adjusted HR per 1 score increase: 1.055, 95% CI: 1.028, 1.083) and death of heart diseases (adjusted HR per 1 score increase: 1.076, 95% CI: 1.021, 1.135) after adjusted for age, diabetes diagnosis (without diabetes, undiagnosed diabetes, and diagnosed diabetes), sex, educational level, BMI, smoking, hypertension, hyperlipidemia, and alcohol consumption. When treated as categorical variable, medium and high DII scores were both correlated with increased risk of all-cause mortality (adjusted HR for medium DII: 1.194, 95% CI: 1.068, 1.336. Adjusted HR for high DII: 1.253, 95% CI: 1.115, 1.407) compared to participants with low DII scores. Moreover, higher HEI scores were associated with lower risk of all-cause mortality (adjusted HR per 1 SD increase: 0.903, 95% CI: 0.859, 0.950) (SD: standard deviation) and death of heart diseases (adjusted HR per 1 SD increase: 0.891, 95% CI: 0.800, 0.992) after adjusting for potential confounders. When treated as categorical variable, high HEI score was correlated with decreased risk of all-cause mortality (adjusted HR: 0.804, 95% CI: 0.708, 0.913) compared with low HEI score. These association remains similar trends in participants with different diabetes diagnosis (*p* for interaction >0.05, supplementary Table S4).

## Discussion

In our study, we systematically estimated the prevalence of diabetes in the general US population, both diagnosed and undiagnosed, as well as the trends in dietary patterns by diabetes diagnoses over the previous decades. Moreover, we investigated the connection between dietary patterns and long-term mortality.

Diabetes control is an urgent issue in American [29]. According to a NHANES statistics in 2021, the prevalence of diabetes in American has significantly increased from 1999–2000 to 2017–2018, with a 3.3 percent relative increase per 2-year [30]. Similar findings were made in our study, which discovered that the prevalence of diagnosed diabetes increased from 8.43% in 2007–2008 to 11.65% in 2017–2018. Notably, the prevalence of undiagnosed diabetes ranged between 3.90% and 5.13% in the overall US adults. Type 2 diabetes has a long asymptomatic phase (5–6 years) before being diagnosed, during which time micro- and macrovascular complications can begin to develop [31–33]. Therefore, it is critical to understand the characteristics of the undiagnosed diabetes population in order to aid in early diagnosis and intervention.

Diabetes care and prevention is a comprehensive endeavor containing both pharmacological and non-pharmacological management. Moreover, diet and lifestyle intervention are even more effective than metformin [34]. HEI was a measure of overall dietary quality, with a higher score indicating better compliance with the American dietary Guidelines for Americans (DGAs). In comparison to participants with undiagnosed diabetes, our study’s findings showed that participants with diagnosed diabetes had significantly higher HEI scores, which are primarily caused by certain food factors like whole grains, fatty acids, and—most importantly—added sugars. The improved HEI scores among participants who had been diagnosed with diabetes demonstrated the value of lifestyle management in the treatment of diabetes, as well as the necessity of early detection and simultaneous intervention in patients with diabetes. Additionally, we noticed a downward trend in HEI scores in each of the three groups over the past few years, which may point to a decline in the efficacy of dietary interventions. Actually, together with the increased prevalence of diabetes, glycemic control in adults with diabetes in the United States has declined since 2010 [29, 30]. The publication of three significant trials in 2008–2009 that demonstrated intensive glycemic control had no cardiovascular profit and increased the risk of hypoglycemia may be a contributing factor in the deterioration of diabetes control [35–37] [38]. Along with worsening glycemic control, cardiometabolic risk factor control in US adults with diabetes is deteriorating [30] [39], which was consistent with the high ratio of hypertension and hyperlipidemia in individuals with diabetes in our findings.

According to the survival analysis in our study, higher scores of HEI was correlated with a lower risk of all-cause mortality and death of heart diseases, which was independent of diabetes diagnosis and other conventional risk factors. This results indicates that a better adherence of dietary guideline may improve the life expectancy in the general Americans. Similarly, higher DII scores were significantly associated with a higher risk of all-cause mortality and death of heart diseases. DII has been demonstrated to be correlated with some chronic metabolic diseases, including obesity, diabetes, and cardiovascular disease, since it was first reported in 2014 [16, 17]. In comparison to participants without diabetes, our study found that participants with diabetes, whether diagnosed or undiagnosed, had higher DII scores. Furthermore, trends in DII scores of general Americans tended to rise after 2010, indicating an increase in dietary inflammatory potential. According to the recommendation of the American Diabetes Association, eating habits such as low-carbohydrate, high non-starchy vegetables, limited refined grains, and added sugars, and choosing whole food over highly processed foods have a positive effect on diabetes [40,41]. Moreover, patients with diabetes were advised to focus on healthy dietary patterns instead of individual foods or nutrients [40]. In light of our discovery of a link between DII scores and long-term mortality, a DII-guided dietary adjustment may be considered in future research.

To our knowledge, this is the first study that compared the dietary patterns of undiagnosed and diagnosed diabetes in general Americans, which provides insight into the characteristics of individuals with undiagnosed diabetes and highlights the urgency of early detection and intervention of diabetes. There were several limitations in our study. First, the HEI and DII were calculated using 24-hour dietary recall data, which cannot fully capture daily eating patterns and may alter over time. Second, we reported two dietary pattern indexes in this work; other dietary patterns like Mediterranean diet were not been included due to statistical limitations, and should be compared in future research. Third, due to the lack of Interleukin-6, Tumor Necrosis Factor-alpha, and other inflammatory variables, we were unable to directly estimate the relationship between DII and inflammatory potential.

## Conclusions

In conclusion, dietary management of patients with diabetes is deteriorating in tandem with the rise in diabetes prevalence in the United States. Participants with undiagnosed diabetes had lower HEI scores than those with diagnosed diabetes, indicating a deviation from dietary recommendations. Furthermore, adults with diabetes, whether diagnosed or undiagnosed, had higher DII scores than the control group. Both lower scores of HEI and higher scores of DII were linked to a higher risk of all-cause mortality and death of heart diseases. According to our findings, extra attention should be paid to the underlying dietary issues of persons with undiagnosed diabetes, as well as the dietary inflammatory potential of people with diabetes.

## Supplementary information

Supplementary materials

## Data Availability

All data in this work are available on the NHANES website (https://wwwn.cdc.gov/nchs/nhanes/Default.aspx).
