# Supplementary material for: Trends in dietary patterns over the last decade and their association with long-term mortality in general US populations with undiagnosed and diagnosed diabetes
Source: Nutr Diabetes. 2023 Apr 19;13:5. doi: 10.1038/s41387-023-00232-8 (PMC10115856; doi:10.1038/s41387-023-00232-8)
Supplement: Supplementary file 1 — Supplementary materials [file 41387_2023_232_MOESM1_ESM.pdf]

Figure S1. Flowchart of individual enrollment

NHANES: the National Health and Nutrition Examination Survey.

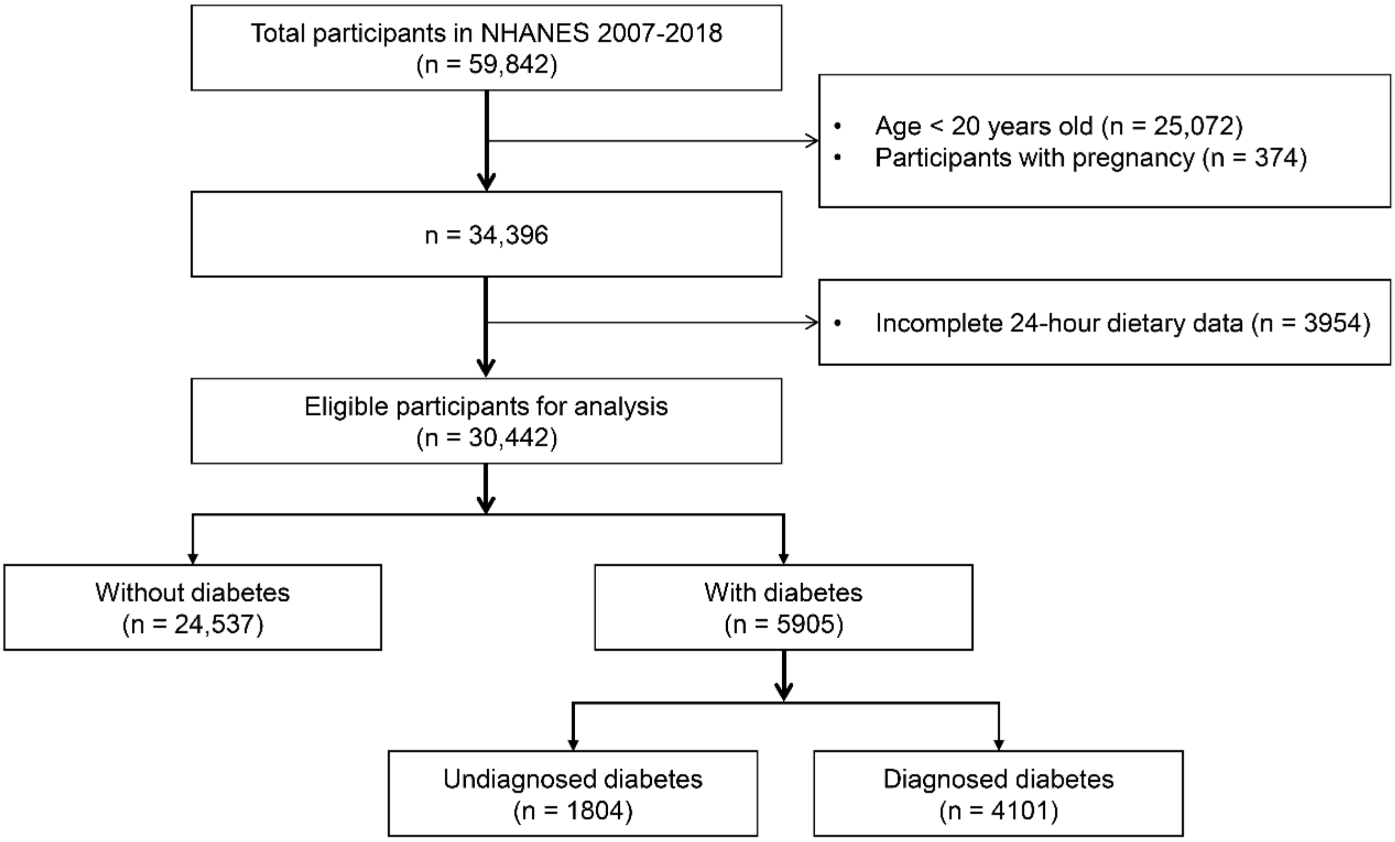

Figure S2. Trends in the prevalence of undiagnosed and diagnosed diabetes in the general American adults

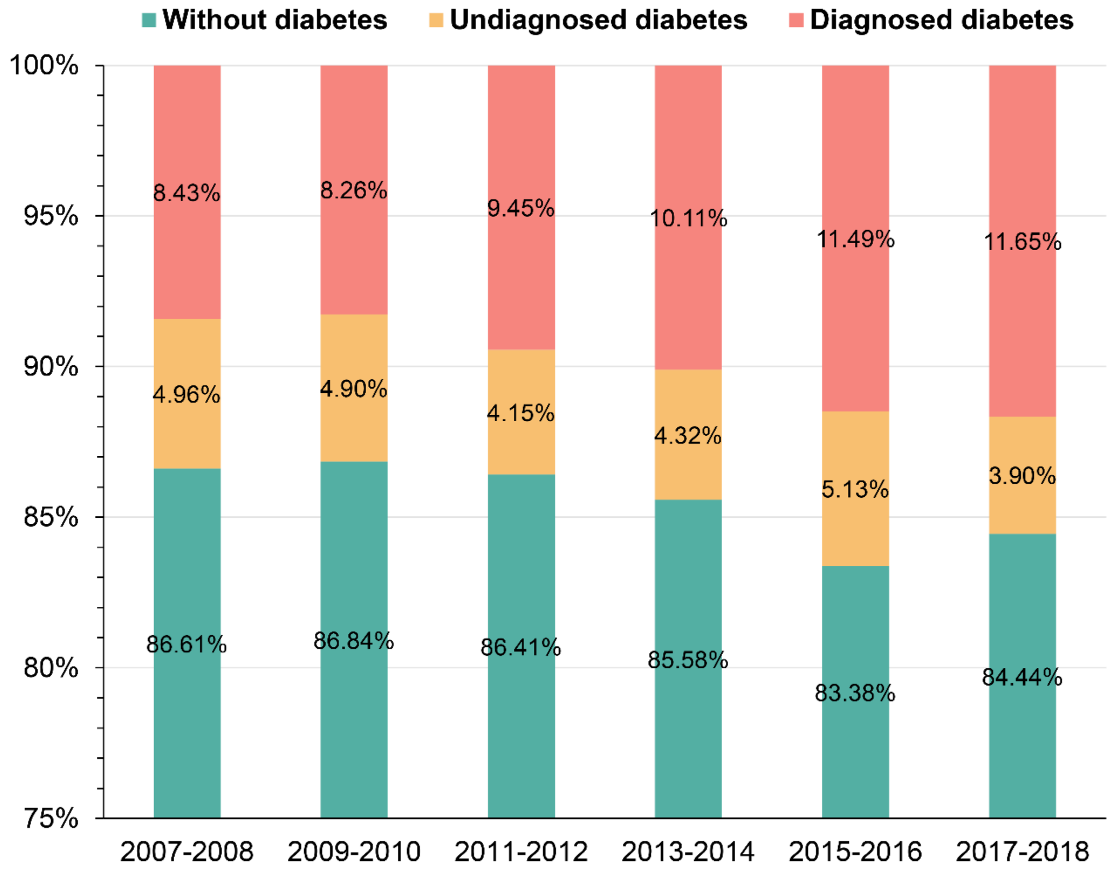

Figure S3. Trends in component scores of healthy eating index scores over the last decade by baseline diabetes diagnosis

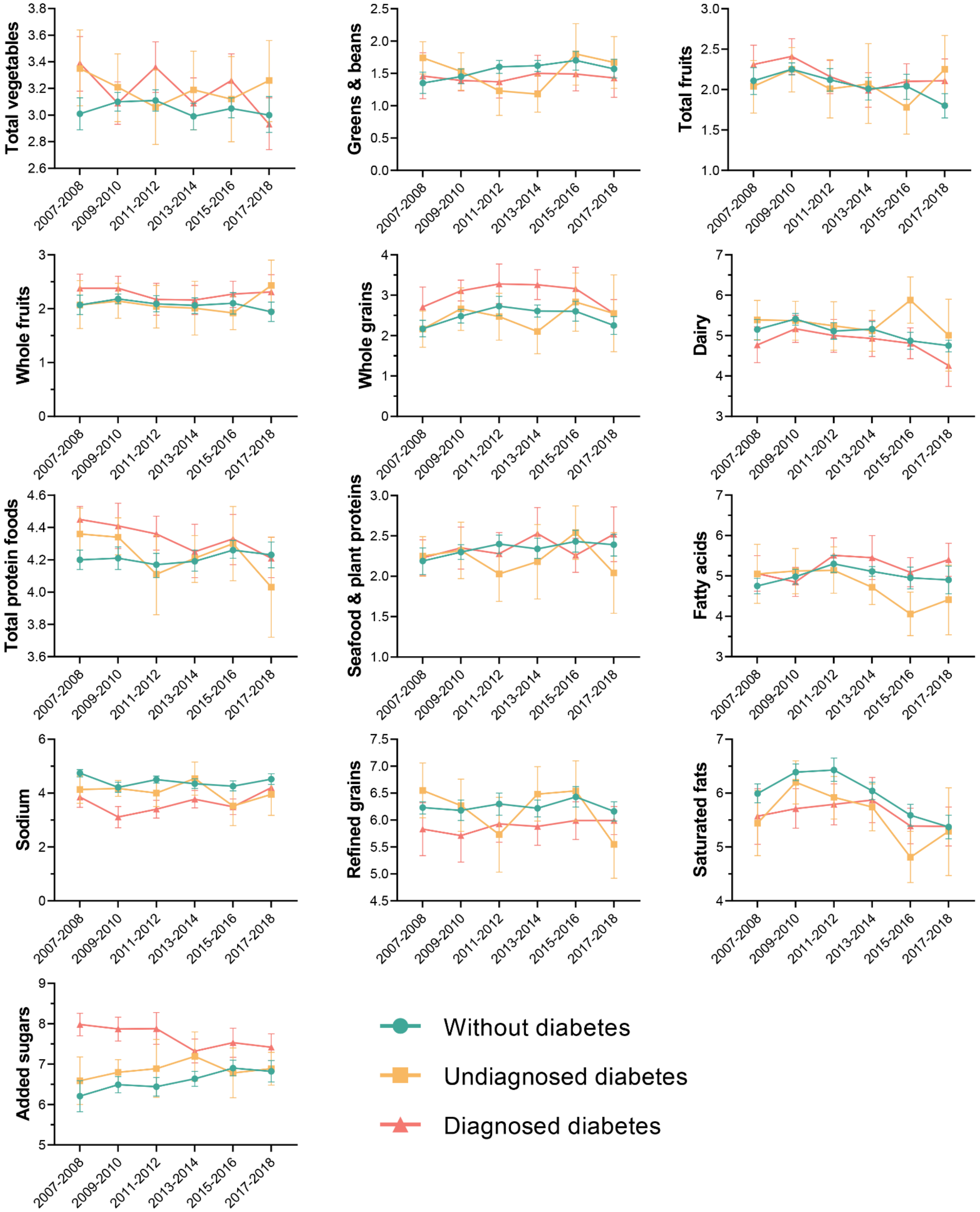

Figure S4. Flowchart of individual enrollment in the survival analysis

NHANES: the National Health and Nutrition Examination Survey.

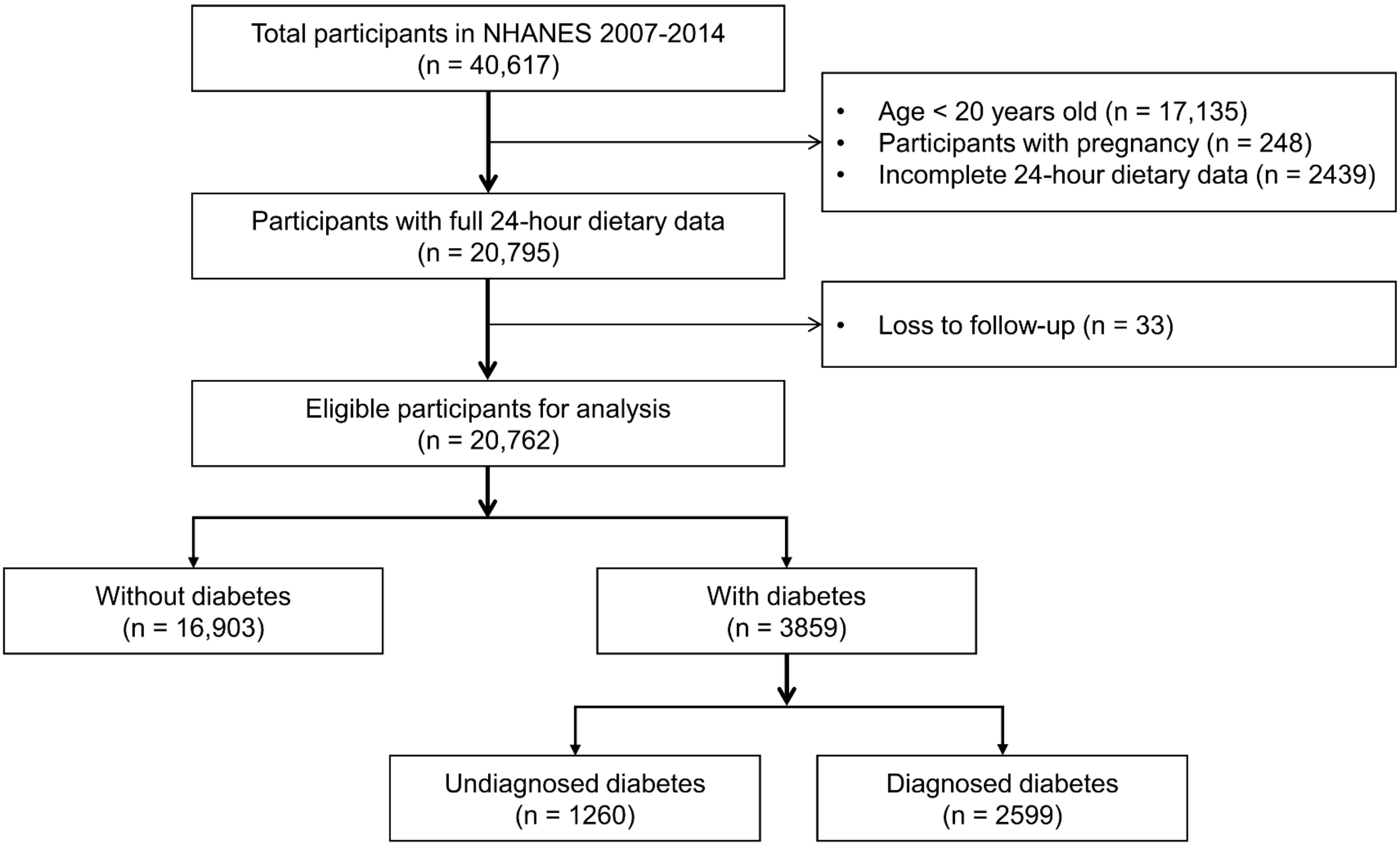

Figure S5. Association between dietary patterns and long-term mortality

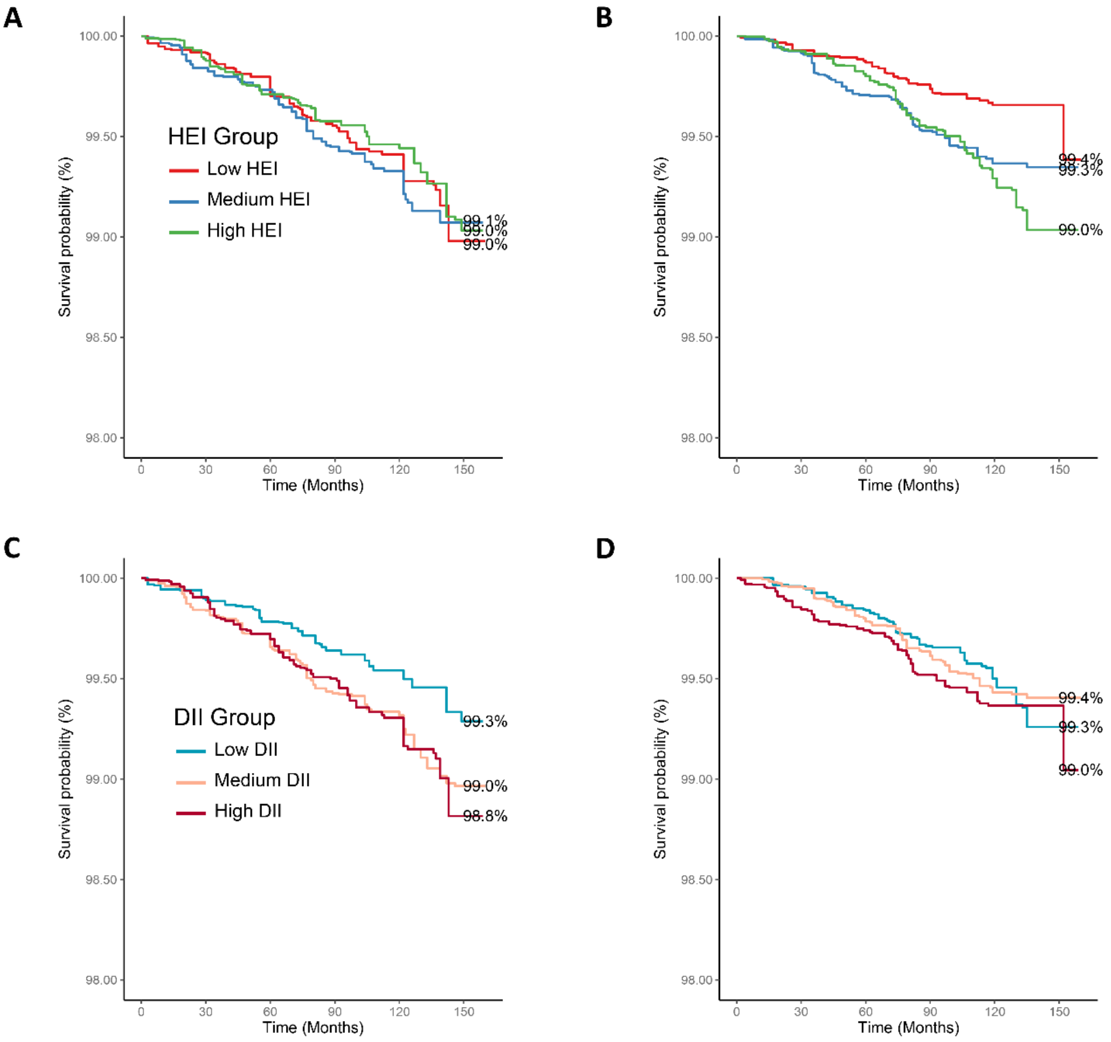

A: Association between HEI and death of chronic lower respiratory diseases; B: Association between HEI and death of cerebrovascular diseases; C: Association between DII and death of chronic lower respiratory diseases; D: Association between DII and death of cerebrovascular diseases

CI: confidence interval; DII: dietary inflammatory index; HEI: healthy eating index; HR: hazard ratio.

Table S1. Maximum scores of each HEI component

Component of HEI	Max score
Whole grains	10
Dairy	10
Fatty acids	10
Refined grains	10
Sodium	10
Added sugars	10
Saturated fats	10
Total fruits	5
Whole fruits	5
Total vegetables	5
Greens & beans	5
Total protein foods	5
Seafood & plant proteins	5

Table S2. Component analysis of HEI scores by baseline diabetes diagnosis

	Without diabetes (n = 24 537)	Undiagnosed diabetes (n = 1804)	Diagnosed diabetes (n = 4101)	*p1*	*p2*	*p3*
Total score of HEI	51.05 (50.56, 51.54)	50.57 (49.79, 51.36)	51.59 (50.93, 52.25)	0.263	0.122	0.038
Total vegetables	3.04 (3.00, 3.09)	3.20 (3.08, 3.32)	3.17 (3.09, 3.25)	0.021	0.004	0.731
Greens & beans	1.55 (1.50, 1.60)	1.54 (1.39, 1.69)	1.44 (1.33, 1.56)	0.889	0.068	0.309
Total fruits	2.05 (1.99, 2.11)	2.06 (1.91, 2.21)	2.16 (2.06, 2.26)	0.932	0.049	0.273
Whole fruits	2.07 (2.01, 2.14)	2.09 (1.92, 2.26)	2.27 (2.16, 2.39)	0.810	0.001	0.084
Whole grains	2.48 (2.39, 2.56)	2.47 (2.21, 2.74)	3.00 (2.82, 3.18)	0.979	< 0.001	0.001
Dairy	5.07 (4.99, 5.15)	5.36 (5.11, 5.61)	4.79 (4.61, 4.97)	0.026	0.007	< 0.001
Total protein foods	4.21 (4.18, 4.24)	4.23 (4.15, 4.32)	4.32 (4.27, 4.38)	0.621	< 0.001	0.079
Seafood & plant proteins	2.34 (2.29, 2.40)	2.24 (2.09, 2.40)	2.37 (2.26, 2.49)	0.220	0.615	0.116
Fatty acids	5.00 (4.90, 5.09)	4.74 (4.48, 4.99)	5.24 (5.07, 5.42)	0.074	0.017	0.001
Sodium	4.43 (4.36, 4.50)	4.04 (3.80, 4.28)	3.67 (3.53, 3.81)	0.004	< 0.001	0.008
Refined grains	6.25 (6.18, 6.32)	6.22 (5.98, 6.45)	5.90 (5.75, 6.05)	0.750	< 0.001	0.029
Saturated fats	5.96 (5.89, 6.04)	5.55 (5.32, 5.77)	5.60 (5.44, 5.76)	< 0.001	< 0.001	0.685
Added sugars	6.59 (6.48, 6.69)	6.85 (6.62, 7.08)	7.63 (7.49, 7.77)	0.040	< 0.001	< 0.001

The data are presented as the mean and 95% confidence interval. HEI: healthy eating index.

p1: participants with undiagnosed diabetes vs. participants without diabetes; *p2*: participants with diagnosed diabetes vs. participants without diabetes; *p3*: participants with undiagnosed diabetes vs. participants without diabetes.

Table S3. Association between dietary index scores and participants' long-term mortality

Variables	Age-adjusted HR (95% CI)	*p1*	Full-adjusted HR (95% CI) *	*p2*
**All-cause mortality**				
HEI (per 1 SD increase)	0.830(0.794,0.867)	< 0.001	0.903(0.859,0.950)	< 0.001
HEI group				
Low HEI	Ref		Ref	
Medium HEI	0.890(0.778,1.019)	0.092	0.991(0.873,1.126)	0.894
High HEI	0.661(0.591,0.740)	< 0.001	0.804(0.708,0.913)	< 0.001
DII (per 1 score increase)	1.074(1.048,1.101)	< 0.001	1.055(1.028,1.083)	< 0.001
DII group				
Low DII	Ref		Ref	
Medium DII	1.236(1.099,1.390)	< 0.001	1.194(1.068,1.336)	0.002
High DII	1.355(1.203,1.525)	< 0.001	1.253(1.115,1.407)	< 0.001
**Death of malignant neoplasms**				
HEI (per 1 SD increase)	0.868(0.791,0.952)	0.003	0.944(0.857,1.039)	0.241
HEI group				
Low HEI	Ref		Ref	
Medium HEI	0.778(0.581,1.042)	0.092	0.856(0.638,1.146)	0.296
High HEI	0.705(0.566,0.880)	0.002	0.858(0.680,1.083)	0.198
DII (per 1 score increase)	1.046(0.989,1.107)	0.114	1.051(0.995,1.110)	0.075
DII group				
Low DII	Ref		Ref	
Medium DII	1.341(1.023,1.758)	0.033	1.343(1.034,1.745)	0.027
High DII	1.189(0.897,1.575)	0.229	1.212(0.932,1.575)	0.152
**Death of heart diseases**				

HEI (per 1 SD increase)	0.805(0.729,0.889)	< 0.001	0.891(0.800,0.992)	0.036
HEI group				
Low HEI	Ref		Ref	
Medium HEI	1.029(0.770,1.376)	0.845	1.155(0.871,1.531)	0.316
High HEI	0.626(0.481,0.815)	< 0.001	0.783(0.597,1.026)	0.076
DII (per 1 score increase)	1.103(1.047,1.163)	< 0.001	1.076(1.021,1.135)	0.006
DII group				
Low DII	Ref		Ref	
Medium DII	1.316(1.033,1.675)	0.026	1.266(1.002,1.599)	0.048
High DII	1.445(1.132,1.845)	0.003	1.297(1.025,1.640)	0.030
**Death of chronic lower respiratory diseases**				
HEI (per 1 SD increase)	0.745(0.614,0.903)	0.003	0.874(0.720, 1.062)	0.176
HEI group				
Low HEI	Ref		Ref	
Medium HEI	0.800(0.496,1.289)	0.359	0.973(0.618, 1.532)	0.907
High HEI	0.478(0.300,0.763)	0.002	0.684(0.433, 1.078)	0.102
DII (per 1 score increase)	1.124(1.012,1.250)	0.030	1.053(0.936, 1.184)	0.390
DII group				
Low DII	Ref		Ref	
Medium DII	1.474(0.864,2.516)	0.154	1.245(0.720, 2.154)	0.434
High DII	1.458(0.857,2.483)	0.165	1.102(0.617, 1.970)	0.742
**Death of cerebrovascular diseases**				
HEI (per 1 SD increase)	0.882(0.732,1.062)	0.185	0.916(0.749,1.120)	0.391
HEI group				
Low HEI	Ref		Ref	
Medium HEI	1.247(0.776,2.004)	0.361	1.360(0.851,2.173)	0.198
High HEI	0.980(0.555,1.730)	0.945	1.103(0.602,2.022)	0.752

DII (per 1 score increase)	1.038(0.922,1.168)	0.540	1.030(0.906,1.171)	0.654
DII group				
Low DII	Ref		Ref	
Medium DII	0.945(0.583,1.535)	0.821	0.920(0.554,1.528)	0.747
High DII	1.035(0.587,1.824)	0.906	1.000(0.549,1.820)	1.000

*Adjusted for age, sex, educational level, BMI, smoke, hypertension, hyperlipidemia, diabetes diagnosis, and alcohol consumption.

CI: confidence interval; DII: dietary inflammatory index; HEI: healthy eating index; HR: hazard ratio; SD: standard deviation.

Table S4. Association between dietary index scores and all-cause mortality by baseline diabetes diagnosis

Variables	Adjusted HR (95% CI) *			*p for interaction*
	**Without diabetes**	**Undiagnosed diabetes**	**Diagnosed diabetes**	
**All-cause mortality**				
HEI (per 1 SD increase)	0.902(0.852,0.955)	1.027(0.864,1.220)	0.873(0.782,0.974)	0.823
DII (per 1 unit increase)	1.041(1.010,1.074)	1.044(0.951,1.147)	1.105(1.041,1.172)	0.068
**Death of malignant neoplasms**				
HEI (per 1 SD increase)	0.923(0.822,1.037)	0.925(0.639, 1.339)	1.055(0.841,1.323)	0.316
DII (per 1 unit increase)	1.042(0.972,1.118)	1.092(0.914, 1.304)	1.051(0.949,1.163)	0.829
**Death of heart diseases**				
HEI (per 1 SD increase)	0.872(0.756,1.005)	0.873(0.684,1.115)	0.937(0.762,1.153)	0.724
DII (per 1 unit increase)	1.075(0.999,1.156)	1.094(0.912,1.314)	1.085(0.974,1.210)	0.833
**Death of chronic lower respiratory diseases**				
HEI (per 1 SD increase)	0.780(0.607, 1.002)	-	0.948(0.671, 1.341)	0.583
DII (per 1 unit increase)	1.109(0.964, 1.276)	-	1.067(0.846, 1.346)	0.376
**Death of cerebrovascular diseases**				
HEI (per 1 SD increase)	0.977(0.776,1.230)	0.824(0.548, 1.239)	0.781(0.578,1.054)	0.226
DII (per 1 unit increase)	0.970(0.849,1.109)	1.026(0.816, 1.291)	1.298(0.965,1.745)	0.044

*Adjusted for age, sex, educational level, BMI, smoke, hypertension, hyperlipidemia, diabetes diagnosis, and alcohol consumption.

CI: confidence interval; DII: dietary inflammatory index; HEI: healthy eating index; HR: hazard ratio; SD: standard deviation.
